# Balanced changes in Ca buffering by SERCA and troponin contribute to Ca handling during β-adrenergic stimulation in cardiac myocytes

**DOI:** 10.1093/cvr/cvu201

**Published:** 2014-09-02

**Authors:** Sarah J. Briston, Katharine M. Dibb, R. John Solaro, David A. Eisner, Andrew W. Trafford

**Affiliations:** 1 Unit of Cardiac Physiology, Manchester Academic Health Science Centre, Core Technology Facility, 46 Grafton St, Manchester M13 9NT, UK; 2 Department of Physiology and Biophysics, University of Illinois at Chicago, Chicago, IL, USA

**Keywords:** Calcium, Buffering, SERCA, Troponin, Phospholamban

## Abstract

**Aims:**

During activation of cardiac myocytes, less than 1% of cytosolic Ca is free; the rest is bound to buffers, largely SERCA, and troponin C. Signalling by phosphorylation, as occurs during β-adrenergic stimulation, changes the Ca-binding affinity of these proteins and may affect the systolic Ca transient. Our aim was to determine the effects of β-adrenergic stimulation on Ca buffering and to differentiate between the roles of SERCA and troponin.

**Methods and results:**

Ca buffering was studied in cardiac myocytes from mice: wild-type (WT), phospholamban-knockout (PLN-KO), and mice expressing slow skeletal troponin I (ssTnI) that is not protein kinase A phosphorylatable. WT cells showed no change in Ca buffering in response to the β-adrenoceptor agonist isoproterenol (ISO). However, ISO decreased Ca buffering in PLN-KO myocytes, presumably unmasking the role of troponin. This effect was confirmed in WT cells in which SERCA activity was blocked with the application of thapsigargin. In contrast, ISO increased Ca buffering in ssTnI cells, presumably revealing the effect of an increase in Ca binding to SERCA.

**Conclusions:**

These data indicate the individual roles played by SERCA and troponin in Ca buffering during β-adrenergic stimulation and that these two buffers effectively counterbalance each other so that Ca buffering remains constant during β-adrenergic stimulation, a factor which may be physiologically important. This study also emphasizes the importance of taking into account Ca buffering, particularly in disease states where Ca binding to myofilaments or SERCA may be altered.

## 1. Introduction

Cardiac contraction is initiated by the systolic Ca transient. The entry of Ca into the cell via the L-type Ca current triggers the release of more Ca from the sarcoplasmic reticulum (SR) through a specialized release channel known as the ryanodine receptor. This process, known as Ca-induced Ca release, is responsible for delivery of Ca to troponin C (TnC) and triggering of contraction. Much work has been devoted to investigating how changes in the properties of the various Ca transporting proteins result in changes in the size and kinetics of the Ca transient and thence of contraction (for reviews see Bers^1^ and Eisner *et al.*^2^).

What is often overlooked is that less than 1% of the Ca that enters the cytoplasm is free with the vast majority being bound to Ca buffers.^3,4^ It is therefore possible that, under some circumstances, changes in the properties of the systolic Ca transient result from changes in buffering properties rather than of the underlying Ca fluxes. Experimentally induced changes in Ca buffering do, indeed, have large effects on the systolic Ca transient.^5^ Recent work has found that Ca binding to cardiac myofibrils is altered in hypertrophic cardiomyopathy.^6–8^ Changes in intracellular pH may also affect Ca binding to buffers.^9^ The major fast cytoplasmic buffers are thought to be TnC and the SR Ca-ATPase (SERCA, predominantly SERCA2a in the myocardium).^3^ Ca binding to these proteins is regulated by β-adrenergic stimulation with phosphorylation of TnI decreasing Ca binding to TnC^10^ and phosphorylation of phospholamban increasing Ca binding to SERCA.^11^ It is therefore likely that the Ca buffering ability of TnC and SERCA will change during β-adrenergic stimulation. To the best of our knowledge, however, no study has attempted to assess whether changes in Ca buffering contribute to alterations of Ca cycling under these conditions. One complication in assessing the contribution of changes in buffering to Ca handling during β-adrenergic stimulation is that there is an accompanying increase in the L-type Ca current and SERCA activity, which will also affect the systolic Ca transient. In this study, therefore, we have investigated the effects of β-adrenergic stimulation on Ca buffering, and used transgenic and knockout mice to distinguish the role played by buffering due to SERCA and troponin. The results show that, in wild-type (WT) mouse ventricular myocytes, β-adrenergic stimulation has no effect on buffering and this reflects a combination of increased buffering by SERCA and decreased buffering by troponin. This constancy of Ca buffering power in the face of β-adrenergic stimulation is likely to be physiologically important.

## 2. Methods

All procedures accord to the UK Animals (Scientific Procedures) Act, 1986 and the University of Manchester Ethical Review Process.

### 2.1 Mice

Phospholamban-knockout mice^12^ (PLN-KO) were obtained from the Mutant Mouse Regional Resource Centers (US, strain Pln Tm1 Egk). Since the PLN-KO mice are homozygous, C57Bl/6J (Charles River, UK) were used as controls. The ssTnI mice are CD-1 strain background in which cardiac troponin I (TnI) has been replaced by the non-protein kinase A (PKA) phosphorylatable slow skeletal troponin (ssTnI).^13^ The colony is heterozygous and therefore their respective wild-type littermates were used as controls. C57Bl/6J mice were used for the thapsigargin study.

### 2.2 Isolation of cardiac myocytes

Mice were killed by cervical dislocation and hearts excised and placed in ice-cold isolation solution. The aorta was cannulated and retrogradely perfused with isolation solution for 10 min at 37°C. Collagenase (0.25–0.5 mg/mL, type I, Sigma, UK) and protease (0.05 mg/mL, type XIV, Sigma, UK) were then added, and the heart digested for 6–7 min. The heart was then perfused with a taurine-containing solution for a further 20 min, and the ventricles were finely minced and filtered through 200 µm gauze to obtain intact cardiac myocytes. Cells were stored in an experimental solution and kept at room temperature.

### 2.3 Solutions

The isolation solution contained (in mM): NaCl, 134; glucose, 11; HEPES, 10; KCl, 4; MgSO_4_, 1.2; and NaH_2_PO_4_, 1.2. The taurine solution contained (in mM): NaCl, 113; taurine, 50; glucose, 11; HEPES, 10; KCl, 4; MgS0_4_, 1.2; NaH_2_PO_4_, 1.2; and CaCl_2_, 0.1. The experimental solution contained (in mM): NaCl, 134; glucose, 11; HEPES, 10; KCl, 4; probenecid, 2; CaCl_2_, 1.0; and MgCl_2_, 1.0. All solutions were titrated to pH 7.34 with NaOH.

### 2.4 Voltage-clamp and measurement of intracellular Ca

Cells were voltage-clamped using the whole-cell patch-clamp technique and stimulated with 50 ms duration pulses from −40 to +10 mV from a holding potential of −60 mV at a frequency of 0.5 Hz using an Axopatch 200B and pCLAMP software.^14^ K^+^ currents were blocked by the addition of 4-aminopyridine (5 mM) and BaCl_2_ (0.1 mM). Electrodes (2–3 MΩ resistance) were filled with (in mM): CsCl, 120; TEA-Cl, 20; HEPES, 10; Na_2_ATP, 5; CsEGTA, 0.02; pH 7.2 with CsOH. All experiments were performed at 37°C. Series resistance compensation, typically 80–90%, was applied to minimize voltage errors during voltage-clamp protocols.

For the PLN-KO studies, the Ca indicator Fluo-5F, pentapotassium salt (Invitrogen, UK; 100 µM) was loaded via the patch pipette and a 475 nm light-emitting diode (Cairn Research Instruments, UK) used to excite the fluorophore. Fluorescence was converted to [Ca^2+^]_i_ using a published method.^4^ Briefly, at the end of the experiment, the cell was damaged with the patch pipette. This resulted in an abrupt increase of [Ca^2+^]_i_ to levels that saturate the indicator, and this was taken as the maximum fluorescence (*F*_max_). Assuming that fluorescence is zero in the absence of Ca then [Ca^2+^]_i_ can be calculated from the level of fluorescence (*F*) as follows:

[Ca2+]i=Kd ⋅F/(Fmax−F)

The value of *K*_d_ was taken to be 1035 nM.^15,16^

For the ssTnI and thapsigargin experiments, the Ca indicator Fura-2 pentapotassium salt (Invitrogen, UK, 100 µM) was used but we were unable to calibrate for Ca and, instead (after subtracting background fluorescence) measured the ratio of the emitted fluorescence excited at 360 nm (*F*_360_) and 380 nm (*F*_380_): *R* = *F*_365_: *F*_380_. The ratio values were normalized to the diastolic value and are expressed as *R*/*R*_rest_, where *R*_rest_ is the ratio at diastolic Ca. In cells where an *F*_max_ measurement was attained, this was found to be 1.98 ± 0.17-fold higher than the caffeine response in isoproterenol (ISO; *n* = 6 cells), showing that the indicator was not saturated.

### 2.5 Calculation of SR content and cytoplasmic Ca buffering

SR Ca content was measured by applying 5 mM caffeine together with 20 mM 2,3-butanedione monoxime (BDM) to release Ca from the SR. BDM was used to prevent excessive cell contraction and also release Ca from the SR.^17^ We also confirmed that BDM application had no effect on Ca buffering (see Supplementary material online, *Figure S1*). For simplicity, in the figures, this caffeine plus BDM solution is referred to as ‘caff’. Measurements were made of both the amplitude of the resulting cytoplasmic Ca transient and the integral of the accompanying Na-Ca exchange (NCX) current. The integral of this current gives a measure of the amount of Ca released from the SR that is pumped out of the cell by NCX.^18^ In order to estimate the total amount of Ca pumped out of the cell, this integral must be corrected for Ca removed by the electroneutral plasma membrane Ca-ATPase (PMCA). As in previous work^19^ we have estimated the fractional contribution of PMCA as follows. The rate constant of decay of the caffeine response (*k*_caff_) gives a measure of the total activity of NCX and PMCA. We then applied Ni (10 mM) to inhibit NCX and then reapplied caffeine. The resulting rate constant (*k*_Ni_) represents PMCA activity. The amount of Ca removed by NCX must be multiplied by *k*_caff_/(*k*_caff_ − *k*_Ni_) to calculate the total amount of Ca pumped out of the cell by the combined effects of NCX and PMCA. This correction factor was identical (*P* > 0.05, *n* = 8-9) between WT (1.53 ± 0.05) and PLN-KO (1.43 ± 0.05). Furthermore, it was unaffected by ISO (*n* = 6–10) in both WT (1.46 ± 0.04) and PLN-KO (1.56 ± 0.05). Once the total Ca ([Ca_T_]) was corrected, we measured calcium buffering as described previously.^4^ Briefly, the calculated total Ca (obtained from the corrected integral) is plotted as a function of the free Ca (from the fluorescent indicator) and fit with a linear regression.

### 2.6 Statistical analysis

Data are presented as mean, ±standard error for *n* experiments. Paired *t*-tests were performed with data normalized where required using either a log_10_ or reciprocal transformation as appropriate for the skew in the data. Differences in data were considered significant where *P* < 0.05.

## 3. Results

**Figure 1:**
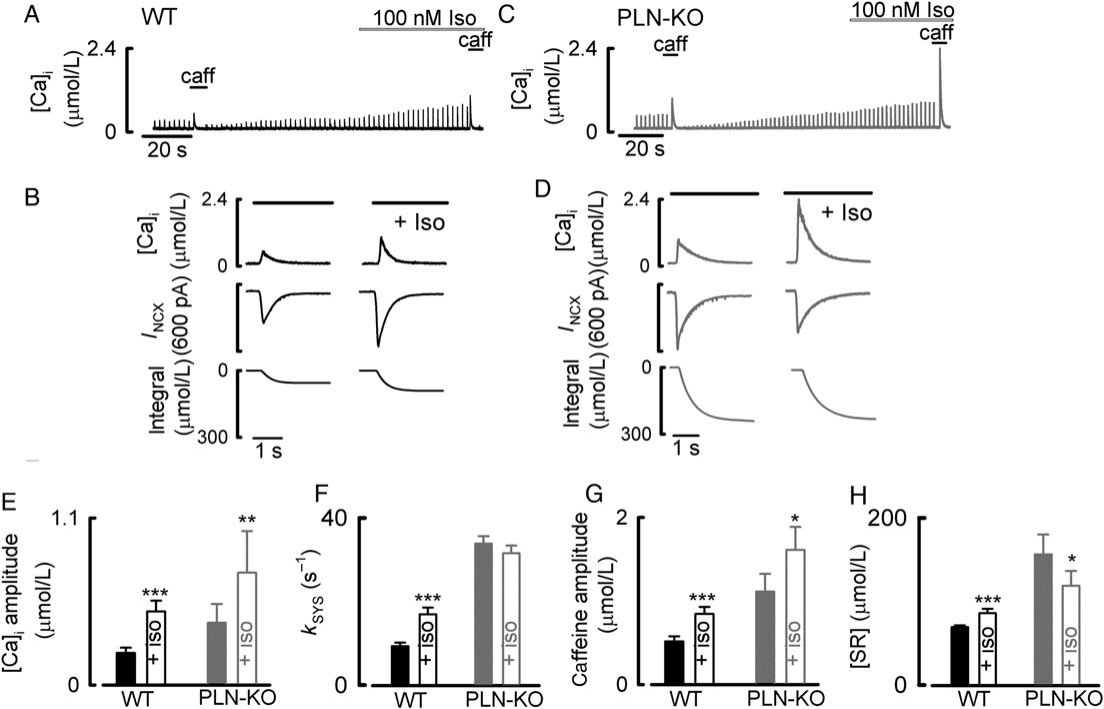
The effects of ISO on Ca signalling in ventricular myocytes from WT and PLN-KO mice. (*A*) Timecourse of effects on a WT cell. The cell was stimulated with voltage-clamp pulses. Caffeine/BDM (caff) and ISO (100 nM) were applied as shown. (*B*) Expanded records of the effects of applying caffeine (5 mM, for the period shown by the horizontal bars) to measure SR Ca content. Traces show (from top to bottom): Ca, membrane current, and integrated current. (*C*) Timecourse of effects on a PLN-KO cell. (*D*) Expanded records of the effects of applying caffeine/BDM to measure SR Ca content. (*E*) Amplitude of the systolic Ca transient. (*F*) Rate constant of decay of the systolic Ca transient. (*G*) Amplitude of the caffeine-evoked increase of Ca. (*H*) Calculated SR Ca content. *P < 0.05; **P < 0.01; ***P < 0.001. PLN-KO: *n* = 8–9 cells/6 animals; WT: *n* = 12 cells/5 animals.

*Figure 1A* shows the results of an experiment designed to investigate the effects of ISO on Ca handling and buffering in WT myocytes. In agreement with previous work,^20,21^ ISO increased the amplitude of the L-type Ca current (not shown) and both the amplitude (*Figure 1E*) and rate constant of decay (*Figure 1F*) of the systolic Ca transient. Caffeine was applied both during control and ISO in order to measure the SR Ca content and to calculate the cellular buffering properties. It is clear from the expanded records (*Figure 1B*) that exposure to ISO increased both the caffeine-evoked increase of [Ca^2+^]_i_ and the accompanying integrated NCX current in WT cells. This is consistent with the reported increase of SR Ca content in response to β-adrenergic stimulation.^14,22–24^ On average (*Figure 1H*), the SR Ca content increased by 24 ± 8% [from 69.2 ± 2.2 to 85.9 ± 5.2 µmol/L (*n* = 12, *P* < 0.001)].

In the results illustrated in *Figure 1C*, a similar experiment was performed on a cell from a PLN-KO mouse. Again, the application of ISO increased the size of the systolic Ca transient (*Figure 1E*) and the calcium current (not shown). As reported previously,^25^ and consistent with the ablation of phospholamban, there was no effect of ISO on the rate constant of decay of the systolic Ca transient (*Figure 1F*). Inspection of the effects of adding caffeine (*Figure 1D*), however, showed differences between PLN-KO and WT cells. ISO increased the amplitude of the caffeine-evoked increase of [Ca^2+^]_i_ by 153 ± 18% (1.1 ± 0.2 to 1.6 ± 0.3 µmol/L, *n* = 8, *P* < 0.05; *Figure 1G*). However, the integral of the NCX-evoked current *decreased* from 156 ± 23 to 119 ± 18 µmol/L (*Figure 1H*). This shows that the total amount of Ca in the SR has been decreased by ISO; however, the increase of free cytosolic Ca when SR Ca is released is greater.

**Figure 2:**
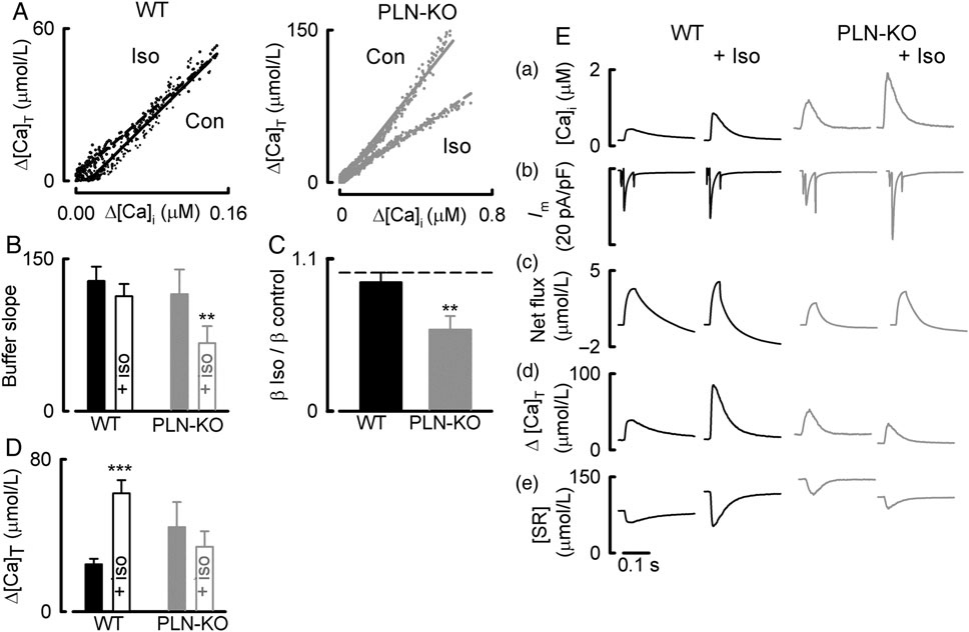
The effects of ISO on Ca buffering in WT and PLN-KO myocytes. (*A*) Buffer curves derived from caffeine-evoked currents. In both panels, data from control and ISO (100 nM) are shown with linear regressions. Left-hand panel shows WT and right-hand PLN-KO. (*B*) Mean data showing the effects of ISO on the buffer slope in WT (left) and PLN-KO (right). (*C*) The effects of ISO on buffer power. The bars show the ratio of buffer power in ISO/buffer power in control for WT (left) and PLN-KO (right). (*D*) The calculated increase of cytoplasmic total Ca during systole. Solid bars, control; open, ISO. (*E*) Quantitative analysis of Ca fluxes. The two left-hand columns are WT and the right-hand PLN-KO myocytes. From top to bottom: [Ca^2+^]_i_; current; net change of cell Ca (calculated from Ca influx and efflux); change of total cytoplasmic Ca; change of SR Ca. **P < 0.01; ***P < 0.001. PLN-KO: *n* = 9–11 cells/6 animals; WT: *n* = 5–12 cells/4–6 animals.

One possible explanation for the opposite effects on free ([Ca^2+^]_i_) and total Ca^2+^ (Ca_T_) would be a change of cytoplasmic Ca buffering power. *Figure 2A* shows the buffering curve calculated from the integrated NCX current.^4^ In the WT myocytes, the slope of this relationship (i.e. the buffering power) was unaffected by β-adrenergic stimulation. In contrast, in the PLN-KO myocytes, there was a marked decrease of buffering power in ISO. *Figure 2B* shows that, on average in WT cells, the buffering power was 128 ± 14 in control and 113 ± 12 in ISO (*n* = 12, *P* > 0.05). In the PLN-KO cells, the slope of the Ca buffer curve (ΔCa_T_/Δ[Ca^2+^]_i_) decreased in ISO from 115 ± 24 to 67 ± 17 (*n* = 9, *P* < 0.01). *Figure 2C* shows that, in PLN-KO cells, the buffer power was reduced to 59 ± 10% of control in ISO. A summary of the effects of ISO on calcium cycling is presented in *Figure 2E* (calculated as described previously^26^). Briefly, net sarcolemmal flux was calculated by integrating the L-type Ca current and the NCX current upon repolarization of the cell, ΔCa_T_ was calculated by converting the changes in free Ca to changes in Ca_T_ using the buffering properties of the cell measured during caffeine application, and changes in SR Ca during the systolic transient were measured by subtracting the change of total cytoplasmic Ca from the net Ca entry into the cell. In both genotypes, ISO increased Ca influx and efflux (*Figure 2E*c). However, the change of *total* cytoplasmic Ca was increased by ISO in WT, but unchanged in PLN-KO (*Figure 2E*d). This was associated with an increase of SR Ca content in WT and a decrease in PLN-KO. *Figure 2D* shows the mean increase of total cytoplasmic Ca during the systolic Ca transient. This was increased by ISO in WT, but was unaltered in PLN-KO.

**Figure 3:**
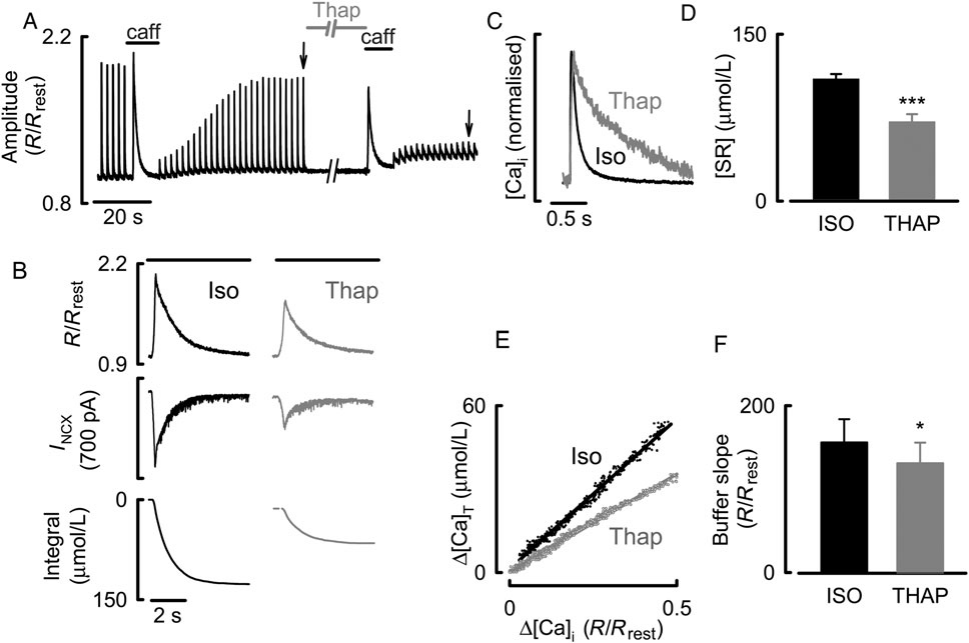
The effects of thapsigargin on Ca handling and buffering. (*A*) Timecourse. The cell was stimulated with voltage-clamp pulses in the presence of ISO (100 nM). Caffeine/BDM (caff) or thapsigargin (Thap, 5 µM) was applied as indicated. Stimulation was stopped during these periods. (*B*) Measurement of SR Ca content and Ca buffering. The three traces show *R*/*R*_rest_, membrane current, and calculated change of total SR Ca. The left-hand panel was obtained in ISO and the right following 90 s exposure to thapsigargin (5 µM), horizontal bars represent the application of caffeine/BDM. (*C*) Specimen, normalized Ca transients obtained at the times shown by arrows in *A*. (*D*) SR Ca content in control (left) and thapsigargin (right). (*E*) The bottom graph shows the buffer curves obtained in ISO and thapsigargin with linear regressions. (*F*) Slope of buffer curve in ISO (left) and thapsigargin (right). *P < 0.05; ***P < 0.001, *n* = 10 cells/3 animals.

Data of *Figures 1* and *2* indicate that the removal of phospholamban reveals an effect of ISO to decrease Ca buffering power. One explanation of this result is that, in WT, activation of SERCA through phosphorylation of phospholamban increases Ca buffering, and that this effect is not seen in PLN-KO animals. The experiment illustrated in *Figure 3* was designed to study whether other approaches to affecting SERCA activity could mimic this. *Figure 3A* shows the effects of the SERCA inhibitor, thapsigargin, on both stimulated Ca transients and caffeine responses. The experiments were performed in ISO (100 nM); thapsigargin (5 µM) was applied for 90 s. In agreement with previous work,^27–29^ and as shown by the normalized traces of *Figure 3C*, thapsigargin slowed the decay of the systolic Ca transient. This was associated with a fall in SR Ca content as shown by the decrease in both the caffeine-evoked increase of Ca and the integral of the accompanying NCX current (*Figure 3B*). On average, the SR Ca content decreased by 35 ± 7% (*Figure 3D*). The calculated buffer curve (*Figure 3E*) shows that Ca buffering was decreased by thapsigargin (*Figure 3F*) and, on average the buffer power decreased by 16 ± 5% [156 ± 28 to 131 ± 25% (*n* = 10, *P* < 0.05)].

**Figure 4:**
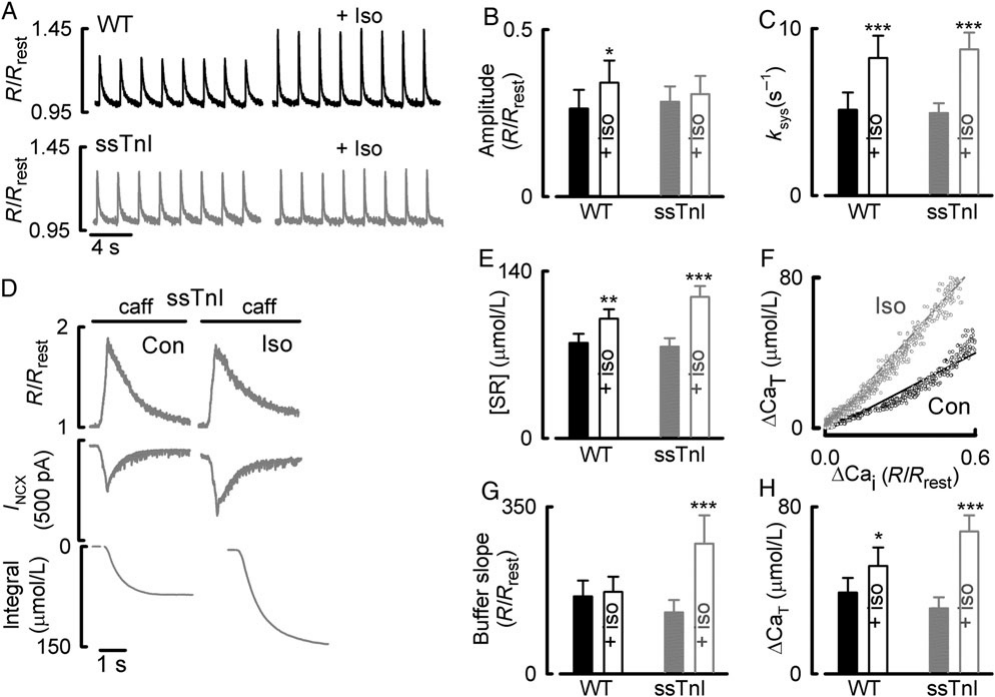
Comparison of the effects of ISO in ssTnI and WT mice. (*A*) The effects of ISO (100 nM) on systolic Ca in WT (top) and ssTnI ventricular myocytes (bottom). (*B*) Mean data for the effects of ISO on the amplitude and rate constant of decay (*C*) of the systolic Ca transient. (*D*) Measurement of SR Ca content and Ca buffering. Traces show (from top to bottom): *R*/*R*_rest_, membrane current, and change of total Ca. The horizontal bars represent the application of caffeine/BDM (caff). (*E*) The effects of ISO on SR Ca content WT (left) and ssTnI (right). (*F*) Buffer curves from control (Con) and ISO with linear regressions. (*G*) The effects of ISO on the buffer slopes in WT (left) and ssTnI (right). (*H*) Mean data for the effects of ISO on the change in Ca_T_ for WT (left) and ssTnI (right). *P < 0.05; **P < 0.01; ***P < 0.001. WT: *n* = 8 cells/6 animals; ssTnI: *n* = 10 cells/5 animals.

The simplest interpretation of the effect of thapsigargin on buffering power is that SERCA contributes significantly to calcium buffering during the systolic Ca transient. We hypothesized that ISO normally increases SERCA activity and Ca binding thereby increasing Ca buffering. The fact that ISO *decreases* buffer power in PLN-KO mice (where this SERCA-dependent effect would not be present) suggests that some additional action of ISO must decrease Ca buffering. The next experiments were designed to investigate whether this additional effect could be related to phosphorylation of TnI. In this work, we compared the effects of ISO on myocytes from mice in which TnI had been replaced with the ssTnI isoform, lacking the PKA phosphorylation site.^13^ *Figure 4A* and *B* shows that ISO had no effect on the amplitude of the systolic Ca transient in ssTnI, but that the rate constant of fall of the systolic Ca transient increases in myocytes from both WT and ssTnI (*Figure 4C*). The results of application of caffeine (*Figure 4D*) show that, in the ssTnI myocytes, ISO decreases the increase of free Ca. On average (expressed in fura-2 ratio units), ISO decreased the caffeine-evoked rise of free Ca from 0.61 ± 0.08 to 0.46 ± 0.06 (*n* = 10, *P* < 0.001), but increased that of total Ca from 76.7 ± 7.1 to 118.5 ± 9.0 µmol/L (*n* = 10, *P* < 0.001). Consequently, ISO increased the Ca buffer power to 128 ± 26% (*n* = 10, *P* < 0.001; *Figure 4G*) of control values. Therefore, despite the fact that ISO increased the change in total Ca (*Figure 4H*), the Ca transient amplitude was unchanged in response to ISO, presumably revealing the effect of SERCA to increase Ca buffering during β-adrenergic stimulation.

## 4. Discussion

This study shows, for the first time in intact cells, that β-adrenergic stimulation alters calcium buffering in cardiac cells at two sites. We find that, in WT cells, β-adrenergic stimulation produces two opposing effects on buffering that cancel each other out. There is an increase of buffering due to phosphorylation of phospholamban activating SERCA and a decrease due to phosphorylation of TnI. Consequently, β-adrenergic stimulation decreases Ca buffering in PLN-KO cells and, conversely, increases it in cells missing the normal adult PKA phosphorylatable form of cardiac TnI. As well as being physiologically important, our results stress the importance of taking changes in Ca buffering into account when assessing changes in Ca handling and SR Ca content.

For ease of discussion, we will consider the results obtained in PLN-KO mice first.

### 4.1 Effects of ISO in PLN-KO cells

We found that ISO increased the amplitude of both the L-type Ca current and the Ca transient.^25,30^ ISO *decreased* the SR Ca content as quantified from the integral of the caffeine-evoked NCX current. There was, however, an *increase* of the [Ca^2+^]_i_ signal in caffeine application. This apparent paradox can be explained by the observed decrease of buffering power such that a given release of Ca results in a larger increase of [Ca^2+^]_i_. The most likely candidate for the decrease of buffering power on exposure to ISO is a phosphorylation of TnI, which has been shown to decrease Ca binding as assessed from a fluorescent reporter attached to TnC.^10^

Analysis of the present data (*Figure 2E*) shows that, in PLN-KO cells, ISO has no effect on either the amplitude of the increase of *total* Ca during systole or on the amount of Ca released from the SR. The increase of the amplitude in the systolic Ca transient is therefore mainly due to the decrease of Ca buffering with a small contribution from the increased L-type Ca current. These results emphasize that the changes in Ca buffering power must be taken into consideration when interpreting changes in [Ca^2+^]_i_.

#### 4.1.1 How does ISO decrease the SR Ca content in PLN-KO cells?

There are three possible explanations. (i) At first sight one might expect, as suggested previously,^25^ that the increased L-type Ca current would load the SR with Ca . However, it should also be noted that an increase of L-type Ca current will also increase the fraction of the SR Ca that is released thereby increasing the amount pumped out of the cell on NCX and tending to decrease the SR Ca content. Indeed, in previous work, we found that increasing the Ca current (by increasing external Ca) either had no effect or *decreased* SR Ca content.^31^ While this mechanism goes in the right direction, it should be noted that a relatively large increase of L-type current had only a small effect on SR content.^31^ It is therefore unlikely that the modest increase of L-type current produced by ISO can account for the marked decrease of SR content. (ii) An increased SR Ca leak could contribute to the lower SR Ca content measured.^32^ We do see an increase in diastolic Ca in the presence of ISO. However, one could argue that an increased SR leak could also lower diastolic Ca due to the increase in NCX-mediated Ca extrusion in the subsarcolemmal space and increased Ca uptake by SERCA. (iii) A more likely explanation is based on the requirement that the cell be in Ca flux balance on each beat. In the steady state, Ca influx and efflux on each beat are equal. The immediate effect of a decrease in Ca buffering would be that a given release of Ca from the SR will produce a larger Ca transient. This will result in more Ca being pumped out of the cell, mainly by NCX, and therefore Ca efflux will be greater than influx tending to decrease SR Ca. A new steady state will be reached when SR Ca has fallen sufficiently that the combination of reduced Ca release from the SR and decreased cytoplasmic Ca buffering result in the same size Ca transient as in control, a situation similar to the application of low-dose caffeine.^33^

The present work may also be relevant to the findings of a previous study that investigated the effects of ISO on SR Ca content in mice, which expressed a non-phosphorylatable version of phospholamban.^34^ That study reported that ISO increased SR Ca content. The increase, however, was measured from the amplitude of the caffeine-evoked increase in [Ca^2+^]_i_ and this may also result from a decrease in Ca buffering rather than an actual increase in the amount of Ca released. This emphasizes the limitations of using the caffeine-evoked increase in [Ca^2+^]_i_ (as opposed to the integral of the NCX current) as a measure of SR Ca content.

One aspect of the present results is difficult to explain. We find no effect of ISO on the rate constant of decay of either the systolic or caffeine-evoked Ca transient in PLN-KO cells. The observed decrease in Ca buffering would be expected to increase the rate constant of decay of both the systolic and caffeine-evoked Ca transients. We have no explanation for this.

### 4.2 Effects of ISO in cells with non-phosphorylatable TnI

In these cells, ISO increased the buffer power. The simplest explanation of this finding is that, in the absence of the normal cardiac TnI, the main effect of ISO is a SERCA-mediated increase of buffering which (in WT) is normally opposed by a decrease of Tn-dependent buffering. This increase of buffering may account for the fact that ISO does not increase the amplitude of the systolic Ca transient in these cells.

### 4.3 Effects of ISO in WT cardiac myocytes

ISO had no effect on Ca buffering in WT cells. There is no doubt that troponin is phosphorylated in WT mice, and that this decreases the Ca sensitivity of contraction.^35,36^ Furthermore, there is no reason to think that the properties of troponin will be different between WT and PLN-KO mice. A more likely explanation is that, in the WT myocytes, the decrease of Ca buffering by phosphorylation of troponin is compensated for by an increase of buffering due to some other component which is absent in the PLN-KO mouse. Thus, the most likely explanation appears to be the phosphorylation of phospholamban, which increases the Ca affinity for SERCA and therefore increases the buffering power. SERCA is generally thought of as a pump that removes Ca from the cytoplasm. However the concentration of Ca-binding sites on SERCA (∼50 µmol/L) makes it quantitatively the second most significant buffer (after troponin, ∼70 µmol/L—see Bers^1^ for values). In this context, a high degree of overexpression of SERCA has been shown to decrease the amplitude of the Ca transient, an effect attributed to Ca buffering.^37^ (see also Higgins *et al.*^38^ for a discussion of the effects of SERCA as a Ca buffer). Therefore, increasing the affinity of SERCA for Ca would be expected to increase Ca buffering. Given that the *K*_d_ for Ca for SERCA is similar to that for troponin (∼0.6 µmol/L^1^), then an increase in buffering due to one would compensate for a decrease due to the other.

It is worth noting that the observation that β-adrenergic stimulation has no net effect on Ca buffering in WT mice may be of physiological significance. It is also experimentally convenient for studies on WT mouse cells since opposing changes in Ca buffering on troponin and SERCA will not affect the overall Ca buffering properties of the cell.

## 5. Study limitations

Our method of determining SR Ca content relies on integration of the NCX current with correction for PMCA-mediated Ca removal. We have not corrected for the contribution of other transporters such as mitochondria. However, mitochondrial and PMCA-mediated Ca efflux only account for <2% of *total* systolic Ca removal.^39^

We have only taken into account the fast cytoplasmic Ca buffers (SERCA and TnC) since although the slow buffers account for the large majority of Ca buffering, they are not particularly relevant with respect to the time-scale that we are measuring.^40,41^

The use of BDM as a contraction uncoupler during caffeine application was necessary, but had no effect on the NCX-mediated Ca decay, determined in sheep myocytes, most likely due to the rapid application and wash out. BDM also had no effects on Ca buffering in these cells as demonstrated in Supplementary material online.

Equipment limitations necessitated the use of different indicators for the PLN-KO and ssTnI studies. At first glance, it appears that the responses to β-adrenergic stimulation are blunted in the ssTnI (*Figure 4B*) compared with the PLN-KO (*Figure 1E*). However, experiments performed using Fluo 5F in both C57 WT and CD-1 WT (background of the ssTnI mice) myocytes showed no differences in response to β-adrenergic stimulation (see Supplementary material online). Since all our data sets are paired (i.e. measurements compared within the same cell) and we are not directly comparing the different strains of mice, then we see no problem with the use of different indicators.

## Supplementary material

Supplementary material is available at *Cardiovascular Research* online.

**Conflict of interest**: none declared.

## Funding

This work was supported by the British Heart Foundation (to K.M.D., D.A.E., and A.W.T.), the Manchester Biomedical Research Centre George Lancashire Award (to A.W.T.), and the US National Institutes of Health grant RO1 HL 022231 and NIH PO1 HL 062426 (to R.J.S.). Funding to pay the Open Access publication charges for this article was provided by the British Heart Foundation awards: FS/12/57, CH/2000/04; PG/11/16 and FS/09/002.
